# Is magnetogenetics the new optogenetics?

**DOI:** 10.15252/embj.201797177

**Published:** 2017-05-23

**Authors:** Simon Nimpf, David A Keays

**Affiliations:** ^1^ Research Institute of Molecular Pathology (IMP)Vienna Biocenter (VBC)ViennaAustria

**Keywords:** Neuroscience

## Abstract

Optogenetics has revolutionised neuroscience as it enables investigators to establish causal relationships between neuronal activity and a behavioural outcome in a temporally precise manner. It is a powerful technology, but limited by the necessity to deliver light to the cells of interest, which often requires invasive surgery and a tethered light source. Magnetogenetics aims to overcome these issues by manipulating neurons with magnetic stimuli. As magnetic fields can pass freely through organic tissue, it requires no surgery or tethering the animals to an energy source. In this commentary, we assess the utility of magnetogenetics based on three different approaches: magneto‐thermo‐genetics; force/torque‐based methods; and expression of the iron chaperone ISCA1. Despite some progress, many hurdles need to be overcome if magnetogenetics is to take the helm from optogenetics.

## Introduction

The pre‐eminent goal in neuroscience is to understand how the brain functions at the anatomical, physiological and molecular level. This aim has been greatly advanced by the development of optogenetics, which enables the manipulation of neuronal activity via light‐sensitive microbial membrane opsins such as channelrhodopsin and halorhodopsin. While this technology has provided unique insights into the circuitry that underlie complex behavioural responses, it has some limitations. Specifically, the necessity to deliver light to the cells of interest often requires invasive surgery; tethering of the animal is frequently necessary; and the heat generated by light‐emitting diodes can cause tissue damage. Magnetogenetics could resolve these issues by activating neurons with a magnetic stimulus as magnetic fields pass freely through organic tissue and could therefore activate any neuronal population no matter its anatomical location without the need for invasive surgery. Ideally, one would exploit the exquisite sensitivity of nature's magnetoreceptors for this purpose.

There are two dominant theories to explain how animals transduce magnetic information into a neuronal stimulus. The first, known as the magnetite theory of magnetosensation, assumes the existence of an intracellular compass composed of magnetite (Fe_3_O_4_) crystals that are coupled to a mechanosensitive channel. Depending on the intensity and/or the polarity of the local magnetic field, the magnetite crystals would exert force on the channel protein, thereby eliciting calcium influx. This contrasts with the light‐dependent hypothesis, which argues that the local magnetic environment influences the spin state of radical pairs in photosensitive molecules, which then alters their biochemical properties.

While co‐opting light‐sensitive molecules for magnetogenetics is likely to be a fruitless exercise, components of a magnetite‐based magnetoreceptor would be valuable. To date, however, the cells and molecules that enable cells to detect magnetic fields remain unknown, and the validity of the magnetite‐based hypothesis is indeed uncertain. A number of groups have therefore tried to engineer artificial magnetosensors. These systems fall broadly into three categories: magneto‐thermo‐receptors which exploit radio frequency fields to activate heat‐sensitive channels; force/torque‐based methods that rely on endogenously generated nanoparticles; and the expression of the iron chaperone ISCA1.

## Magneto‐thermo‐genetics

**Figure 1:**
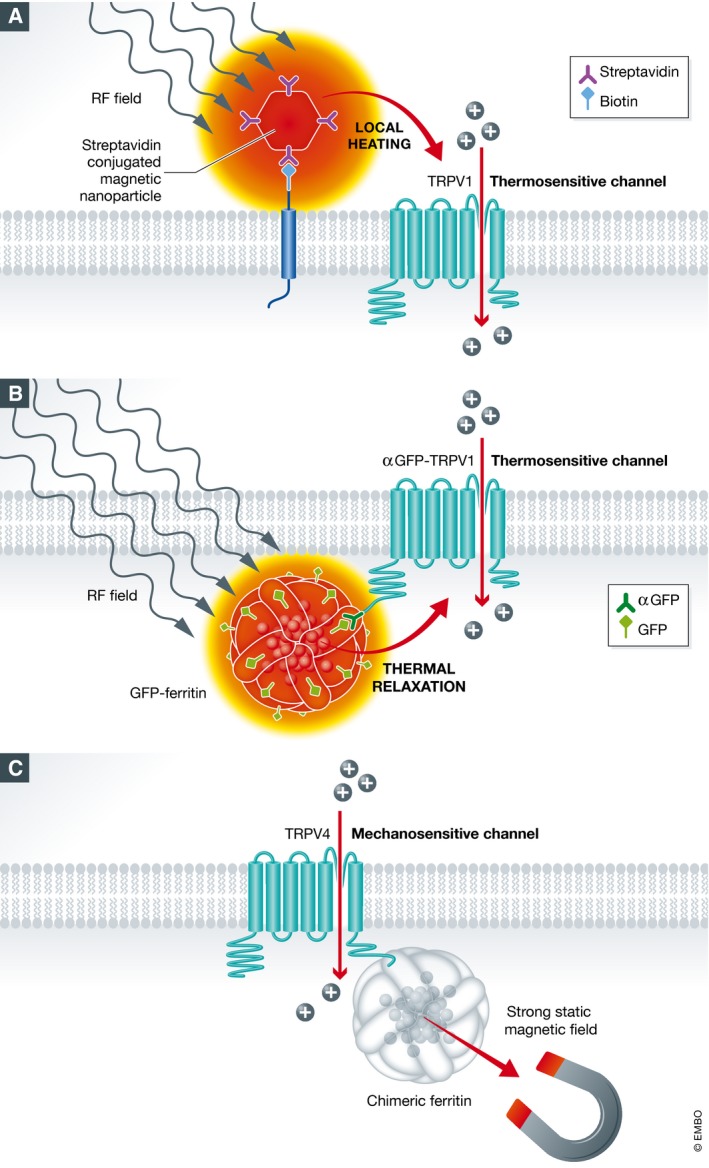
Methods to control the nervous system using magnetogenetics (A) Schematic of the magnetogenetic method developed by Pralle and colleagues (Huang *et al*, 2010). Streptavidin‐conjugated magnetic nanoparticles (MnFe_2_O_4_) are targeted to the cell membrane by a genetically encoded transmembrane domain with a biotinylated biotin acceptor site. Application of an RF magnetic field (40 MHz, 0.84 mT) generates local heating of the nanoparticles, which triggers opening of heterologously expressed thermosensitive ion channels (TRPV1). (B) Schematic of the method developed by Stanley and colleagues, which relies on a genetically encoded ferritin nanoparticle coupled to TRPV1 via a GFP nanobody. It is thought that application of an RF magnetic field (465 kHz, 23–32 mT) leads to thermal relaxation of the central iron core of ferritin and cation influx through the thermosensitive channel (Stanley *et al*, 2016). (C) Schematic of the single‐component magnetogenetic system developed by Wheeler and colleagues. It consists of a chimeric ferritin protein directly coupled to the mechanosensitive cation channel TRPV4. Application of a strong static magnetic field (~50 mT), using an electromagnet, results in calcium transients possibly through a force‐based mechanism (Wheeler *et al*, 2016).

Magneto‐thermo‐genetics relies on a principle known as thermal relaxation, whereby an alternating magnetic field, such as a radiofrequency field, is able to heat up small magnetic nanoparticles. The concept has been widely exploited for cancer therapeutics and more recently adapted for neuronal activation. The key elements are the specific frequency of the magnetic field and the size and composition of the nanoparticles. Pralle and colleagues used manganese oxide nanoparticles (MnFe_2_O_4_) targeted to the cell membrane of human embryonic kidney (HEK) cells to activate the heat‐sensitive TRPV1 channel (Huang *et al*, 2010). A RF stimulus of 40 MHz and 0.84 mT induced thermal relaxation of the nanoparticles, which increased the temperature at the plasma membrane and triggered calcium influx through TRPV1 (Fig 1A). The authors reported that this method evoked action potentials in cultured hippocampal neurons expressing TRPV1.

A similar approach has subsequently been applied to vertebrate systems by the Anikeeva laboratory (Chen *et al*, 2015). They used untargeted polyethylene glycol‐coated synthetic magnetite nanoparticles (Fe_3_O_4_, 22 nm in diameter), a RF stimulus (500 kHz, 15 kA/m) and a TRPV1 transgene to induce calcium influx in HEK cells, action potentials in primary hippocampal neurons and neuronal activation in deep brain areas *in vivo* in mice. The latter, which was assessed by quantitating c‐Fos‐positive cells, was only apparent when both TRPV1 and magnetite nanoparticles were delivered to the brain. While the size and elemental composition of artificial nanoparticles permit the generation of heat with greater precision, the nanoparticles must still be delivered by injection, which risks tissue damage, and their dispersion over time. The ideal system would therefore require genetically encoded nanoparticles.

Stanley and colleagues sought to address this challenge by ectopically expressing chimeric ferritin tethered to TRPV1 via a GFP nanobody (Fig 1B) (Stanley *et al*, 2012, 2016). In vertebrates, the ferritin supercomplex is made of 24 subunits of both light and heavy chains that enclose an iron oxide nanoparticle. This particle, which is ~6 nm in size, is predominantly composed of ferrihydrite (Fe^3+^_10_O_14_(OH)_2_) but may also contain magnetite (Fe_3_O_4_) and maghemite (Fe_2_O_3_) phases (Quintana *et al*, 2004). After validating their construct in cell culture, Stanley *et al* exploited it to manipulate neuronal activity *in vivo*, using an adenovirus to deliver the construct to glucose‐sensing neurons in the ventromedial hypothalamus of mice (Stanley *et al*, 2016). They reported that the application of RF fields (465 kHz, 23–32 mT) increased blood glucose levels dramatically, akin to optogenetic activation. They also showed that this physiological response correlated with c‐Fos activation in hypothalamic neurons expressing the TPRV1‐ferritin construct.

The utility of this technology was expanded further by the creation of an inhibitory TRPV1 channel with a preference for Cl^−^ ions. Delivery of this channel to the hypothalamus of fasted mice resulted in a significant reduction in blood glucose that was only apparent in the presence of RF fields. Intriguingly, Stanley and colleagues have also shown that the application of strong static fields (280 mT to 1 T) induces calcium influx in a hypothalamic cell line, alters the membrane potential and firing rate in slice culture and can influence feeding behaviour of mice if the GFP–TRPV1/GFP–ferritin transgene is present (Stanley *et al*, 2016). This unexpected result raises the prospect that the system actually acts by a force‐based mechanism as TRPV1 is also known to be sensitive to mechanical stimuli.

## Torque‐based magnetogenetics

An alternative to the generation of heat by an oscillating RF field is using a strong magnetic gradient that exerts a force on a magnetic particle. Wheeler and colleagues (2016) developed such a construct by coupling the polymodal cation channel TRPV4 to a fusion of the light and heavy chains of ferritin (Fig 1C). The generation of this construct, which they termed “Magneto2.0”, required some engineering. Most variants were ineffective in a cell culture system, and a plasma membrane trafficking signal was found to enhance its membranous localisation. They report that introduction of this construct into HEK cells results in robust magnetic sensitivity when applying a 50 mT static field, that is blocked by the TRP antagonist ruthenium red (Wheeler *et al*, 2016). They further showed that adenovirus‐mediated delivery of their construct into dopaminergic neurons in the striatum of mice increases the cells’ firing rate when applying a strong static magnetic stimulus (50 mT), which allowed them to manipulate reward‐seeking behaviour in freely moving mice. The mice were placed in a behavioural maze and presented with a choice between two arms; one lined with strong neodymium magnets (50–250 mT) and the other unmagnetised. Animals expressing the Magneto2 construct driven by a dopamine receptor 1 promoter significantly preferred the magnetised arm, in contrast to control animals that exhibited no preference.

## ISCA1 and magnetogenetics

The third magnetogenetic system purports to use the iron chaperone protein ISCA1, but these claims should be viewed with caution (Long *et al*, 2015). This work originated from a recent manuscript that attempted to unite elements of the light‐dependent hypothesis of magnetosensation with an iron‐based mechanism. Based on *in silico* and biochemical methods, Xie and colleagues claim to have discovered a magnetic protein biocompass composed of the light‐sensitive molecule cryptochrome (CRY4) and ISCA1 (Qin *et al*, 2016). It is, however, manifestly unlikely that this complex—if it actually exists in nature—underlies the magnetic sense. ISCA1 is an iron chaperone associated with the biogenesis of iron–sulphur clusters, it binds few iron atoms, has been associated with mitochondrial dysfunction syndrome in humans and is ubiquitously expressed in all cell types in eukaryotes. Nonetheless, Long and colleagues overexpressed pigeon ISCA1 protein in HEK cells, primary hippocampal neurons and *C. elegans* muscle cells (Long *et al*, 2015). They claim that ectopic ISCA1 expression confers sensitivity to a strong static magnetic field (1–2.5 mT) in all cell lines tested. However, these results seem improbable given that all the cells studied already express ISCA1 homologues—as did the control cells—and there is no reasonable mechanistic explanation for ISCA1‐mediated calcium influx. Moreover, a carefully controlled study that employed numerous cell types, calcium indicators and multiple magnetic stimuli failed to replicate any of these findings (Pang *et al*, 2017). Similar attempts to reproduce these results in our laboratory have likewise been unsuccessful. Given the lack of reproducibility and absence of a potential mechanism, ISCA1 does not seem a suitable actuator for magnetogenetics.

## Issues with magnetogenetics (and how we can solve them)

It might appear that optogenetics is on the brink of extinction, about to succumb to human ingenuity and advances in technology. Unfortunately, the current incarnations of magnetogenetics have a number of major issues. First, it is unclear how those that rely on genetically encoded ferritin nanoparticles, actually work. Whether it is a mechanical force or thermal induction, our current knowledge of the ferritin moiety indicates that it lacks the magnetic properties to activate either a mechanical or temperature‐sensitive channel. For instance, the force generated by a single ferritin nano‐particle, which contains about 4,500 iron atoms, in a 50 mT field with a gradient of 6.6 T/m is just 7 × 10^−23^ N, well below the 2 × 10^−13^ N required to open known mechanoreceptors (Meister, 2016). It is therefore important that the studies by Stanley *et al* (2012, 2016) and Wheeler *et al* (2016), both of which were well controlled, are independently replicated. If this confirms that their results are not artefacts, the expertise of biologists and physicists alike will be needed to ascertain the underlying biophysical mechanisms. This will involve studies on the localisation, shape and magnetic properties of the nanoparticle within the ferritin supercomplex, the effects of ferritin clustering around receptors, the elemental composition of the superstructure, the thresholds for receptor activation and whether any electrical induction might be possible.

The second limitation of magnetogenetics in its current form is the infrastructure required for an experiment. The main investment for an optogenetics experiment is the light source, which is inexpensive and simple to apply. A magnetogenetics experiment requires powerful magnetic fields, which are not easy to produce and often risk introducing artefacts. RF generators are expensive, there are few commercially available options, and the coils used to generate the fields produce considerable heat. Similarly, electrophysiological experiments are perilous, because oscillating magnetic fields, by their very nature, induce an electromotive force in conducting electrodes. Static neodymium magnets avoid many of the aforementioned issues, but their application invariably lacks precision, and they can interfere with ferromagnetic components of microscopes. There is still much room for improving coil systems for magnetogenetics, ideally incorporating double wrapping to control for heat and steeper magnetic gradients for torque‐based systems.

The third and less‐publicised issue with magnetogenetics is that it just does not work very well. Current approaches are far less effective than their counterparts in optogenetics, and setting up an experiment, and getting it to work, is an extremely frustrating endeavour. Moreover, activation by magneto‐thermal methods in primary neuronal cell culture is also much slower. Magneto‐thermo‐genetics requires seconds to induce action potentials, whereas optogenetics achieves this within milliseconds.

Efficiency and speed could be improved in a number of ways. To date, most genetically encoded systems use a fusion protein of human light and heavy‐chain ferritin. It is apparent, however, that various mutant forms of ferritin are able to load more iron and consequently have a greater magnetic susceptibility. Jasanoff and colleagues have recently conducted a mutagenesis screen on heavy‐chain ferritin from the thermophilic bacterium *Pyrococcus furiosus*, and identified a mutant (L55P) that triples iron loading, which dramatically improves the capture of cells by high‐gradient magnetic cell separation (Matsumoto *et al*, 2015). There is also scope to co‐opt ferritins from species known to generate magnetite, such as chitons, to further enhance the magnetic properties of the system. Similarly, future incarnations of magnetogenetic sensors may incorporate temperature‐ or mechano‐sensitive channels with lower thresholds of activation, such as TRP channels from infrared‐sensing snakes or vampire bats.

## Concluding remarks

An ideal magnetogenetic system would rely on an inducible, genetically encoded molecule that enables temporally precise and robust activation of neurons wherever they are located and in any species. Such a system might eventually rival optogenetics as the pre‐eminent tool in neuroscience; at present, however, there are many technical obstacles in the way. Deciphering how animals detect magnetic fields could be a critical game changer, but that too remains a daunting challenge. Either way, it is clear that developing robust and reliable sensors for magnetogenetics is not an easy task. Like the early days of optogenetics, magnetogenetics is an arena for ambitious scientists, whose perseverance is destined to be tested as they strive to develop a tool that is both simple and potent. Is magnetogenetics the new optogenetics? The short answer is no, but it might be some day.

## Conflict of interest

The authors declare that they have no conflict of interest.
